# Supplementary material for: Prediction of biomarkers and therapeutic combinations for anti-PD-1 immunotherapy using the global gene network association
Source: Nat Commun. 2022 Jan 10;13:42. doi: 10.1038/s41467-021-27651-4 (PMC8748689; doi:10.1038/s41467-021-27651-4)
Supplement: Supplementary file 1 — Supplementary Information [file 41467_2021_27651_MOESM1_ESM.pdf]

Supplementary Figures and Tables

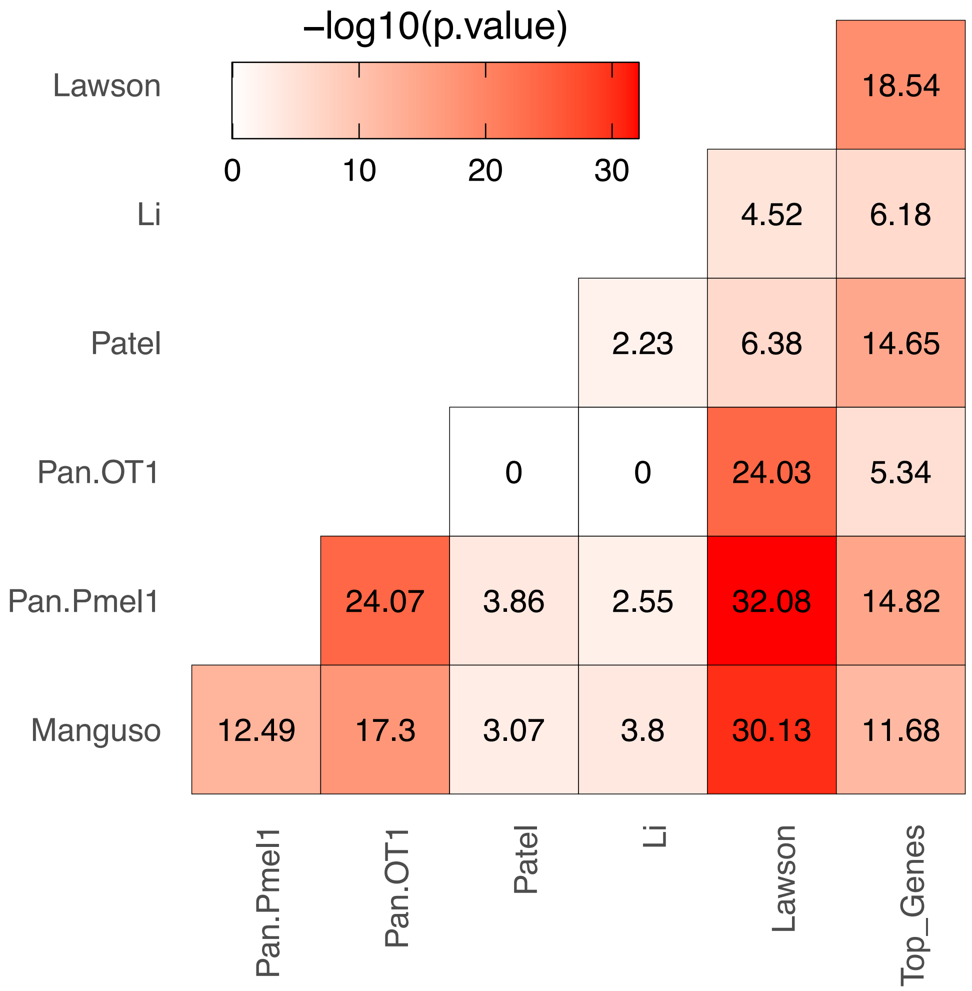

Supplementary Figure 1: Heat map showing the statistical significance of the pairwise overlap analysis of the top 10% of genes in our MHC I-association prediction list (denoted Top_Genes) and the 6 CRISPR-based gene sets (same denotations as Fig. 2). The color scale in the heat map graph indicates the statistical significance of the overlap, –log10(*p*-value), calculated by a one-sided hyper-geometric test. The source data of this figure is provided in the Source Data file.

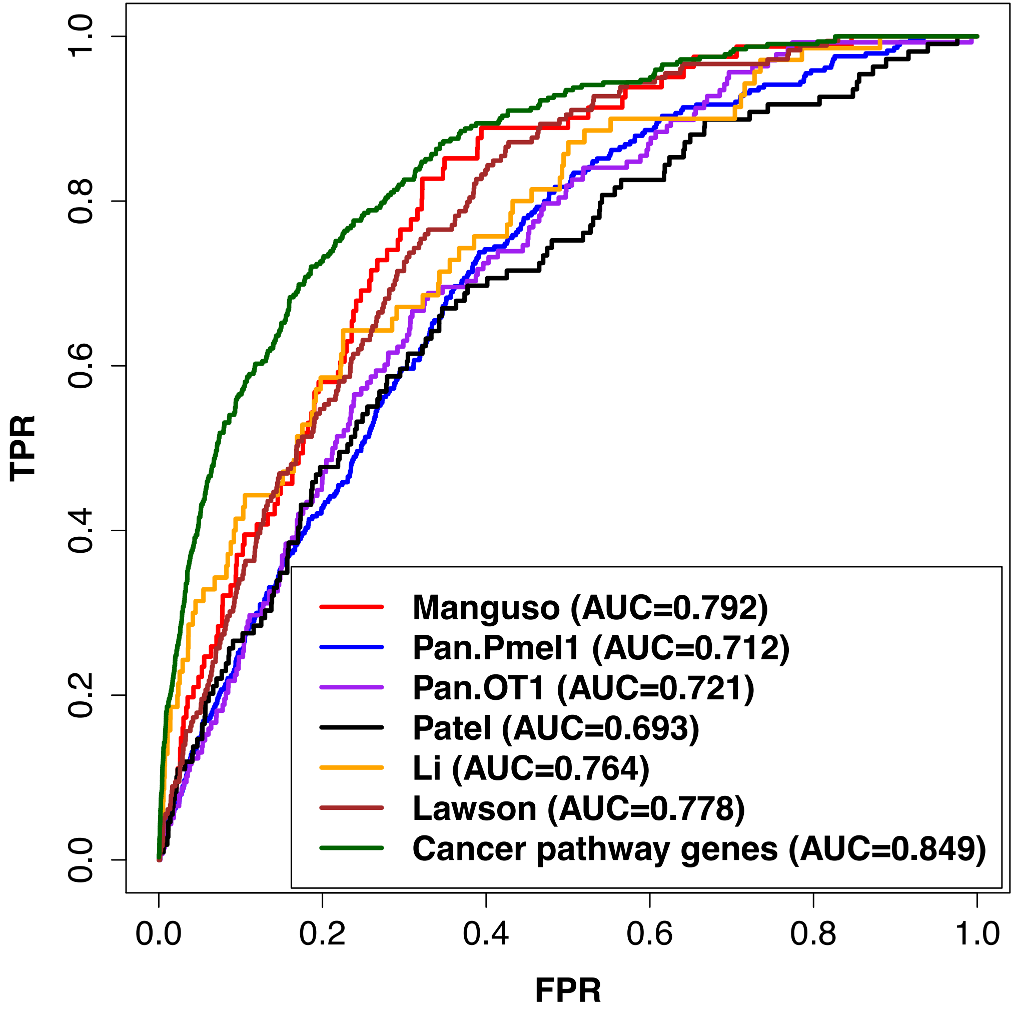

Supplementary Figure 2: Evaluation of predictions generated by the betweenness centrality method using the 6 CRISPR-based gene sets and the KEGG cancer pathway gene set. The gene sets are indicated as in Fig 2. The source data of this figure is provided in the Source Data file.

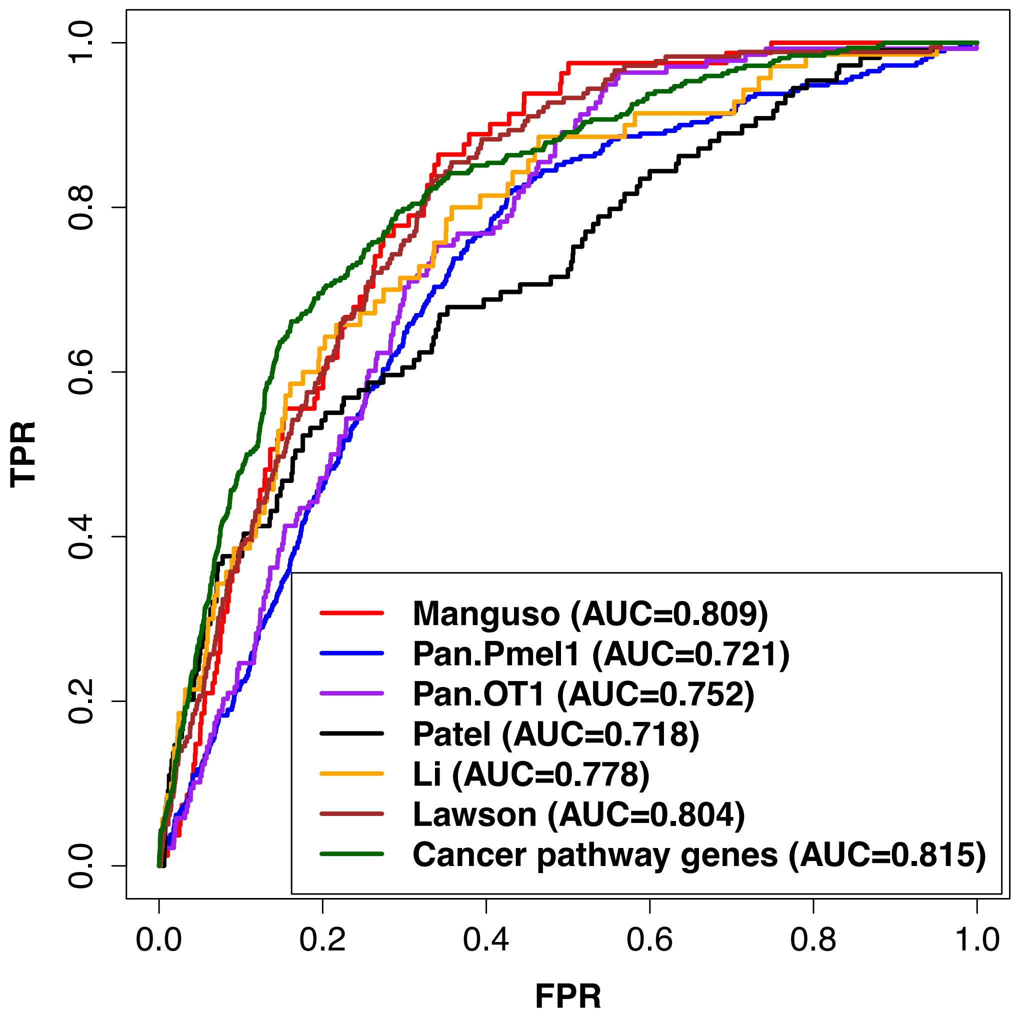

Supplementary Figure 3: Evaluation of predictions generated by the eigenvector centrality method using the 6 CRISPR-based gene sets and the KEGG cancer pathway gene set. The gene sets are indicated as in Fig 2. The source data of this figure is provided in the Source Data file.

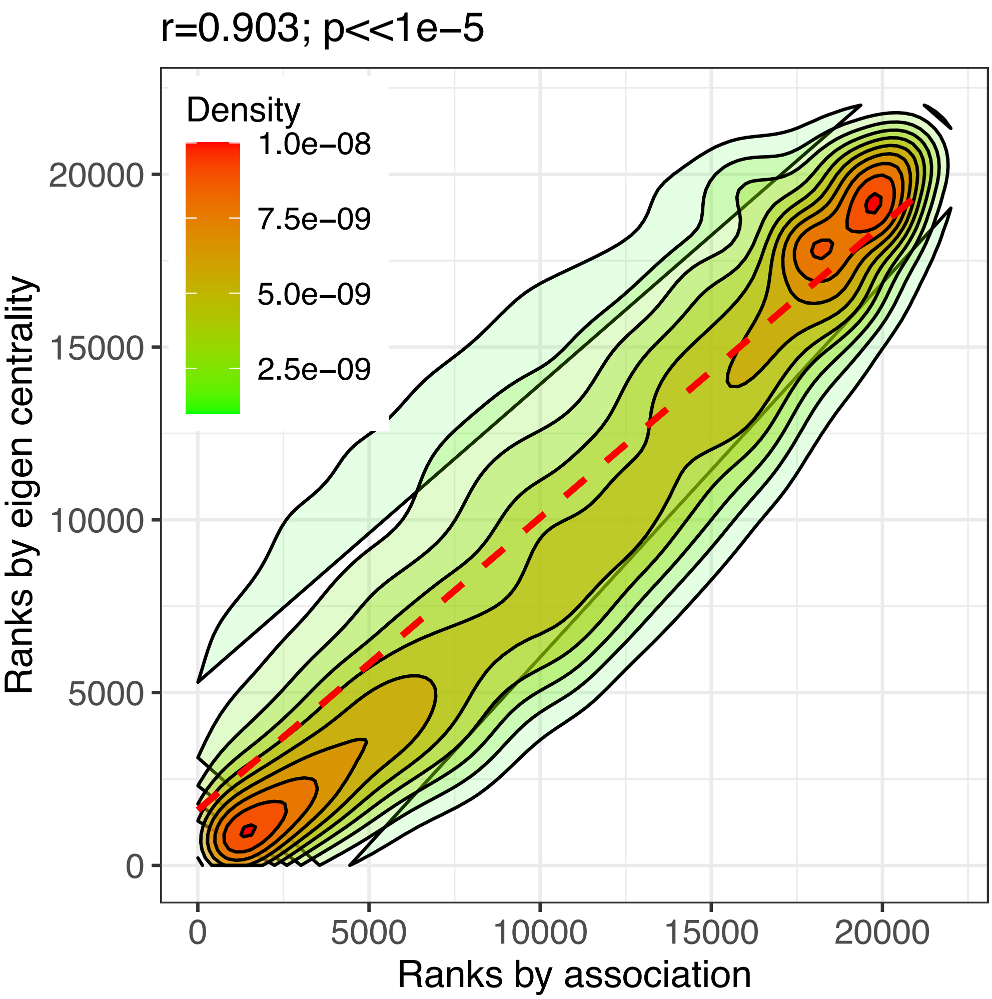

Supplementary Figure 4: Spearman‘s rank correlation analysis of the prediction generated through MHC I association and the prediction generated using the eigenvector centrality method. The correlation coefficient and p-value of the analysis is shown in the upper corner of the plot. The source data of this figure is provided in the Source Data file.

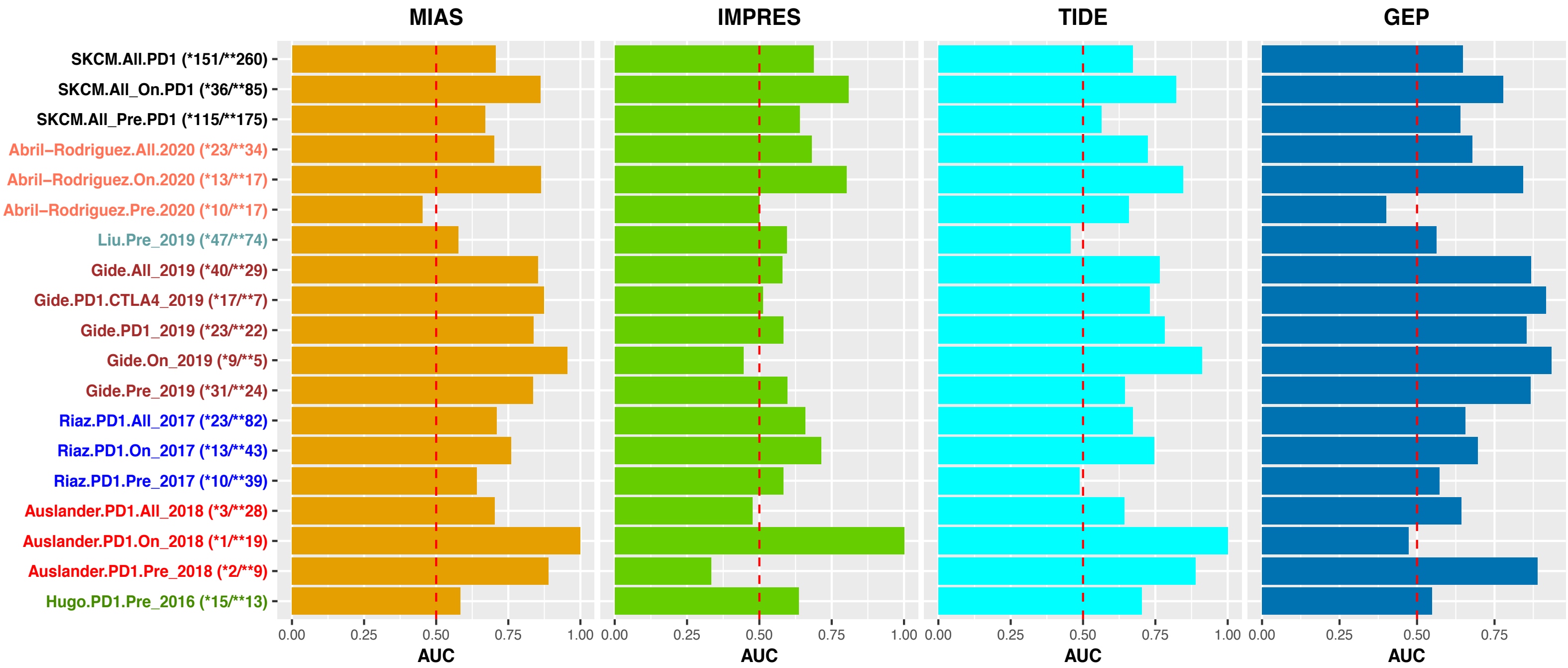

Supplementary Figure 5: Performance comparison of our approach with three prior methods, IMPRES, TIDE and GEP, using area under the curve (AUC) values of receiver operating characteristic (ROC) curves across several melanoma patient cohort data sets. The dotted line in the bar plots represents AUC = 0.5. The source data of this figure is provided in the Source Data file.

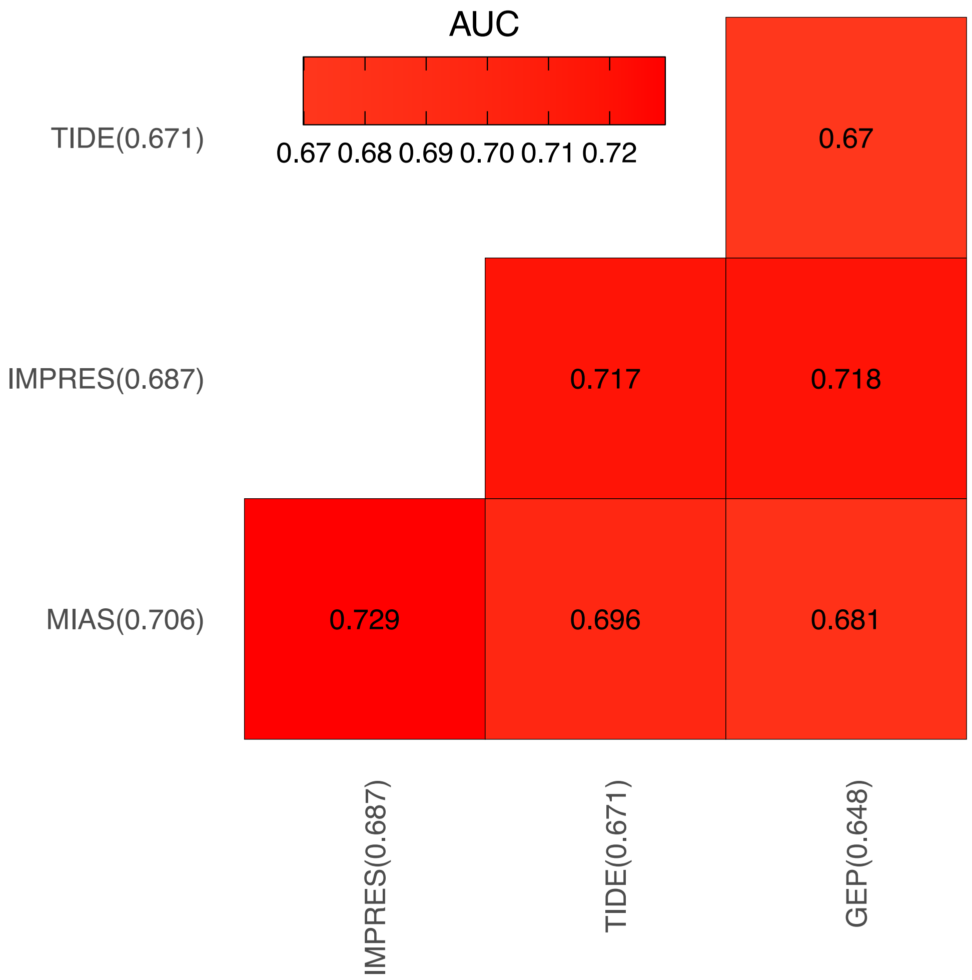

Supplementary Figure 6: Performance of pairwise integrated predictions of MIAS and the three prior methods, IMPRES, TIDE and GEP, for the combined dataset (merged the datasets from all cohorts). The color scale in the heat map graph indicates the statistical significance of prediction performance, AUC. The AUC value of each individual method was also listed inside parentheses behind its name. The source data of this figure is provided in the Source Data file.

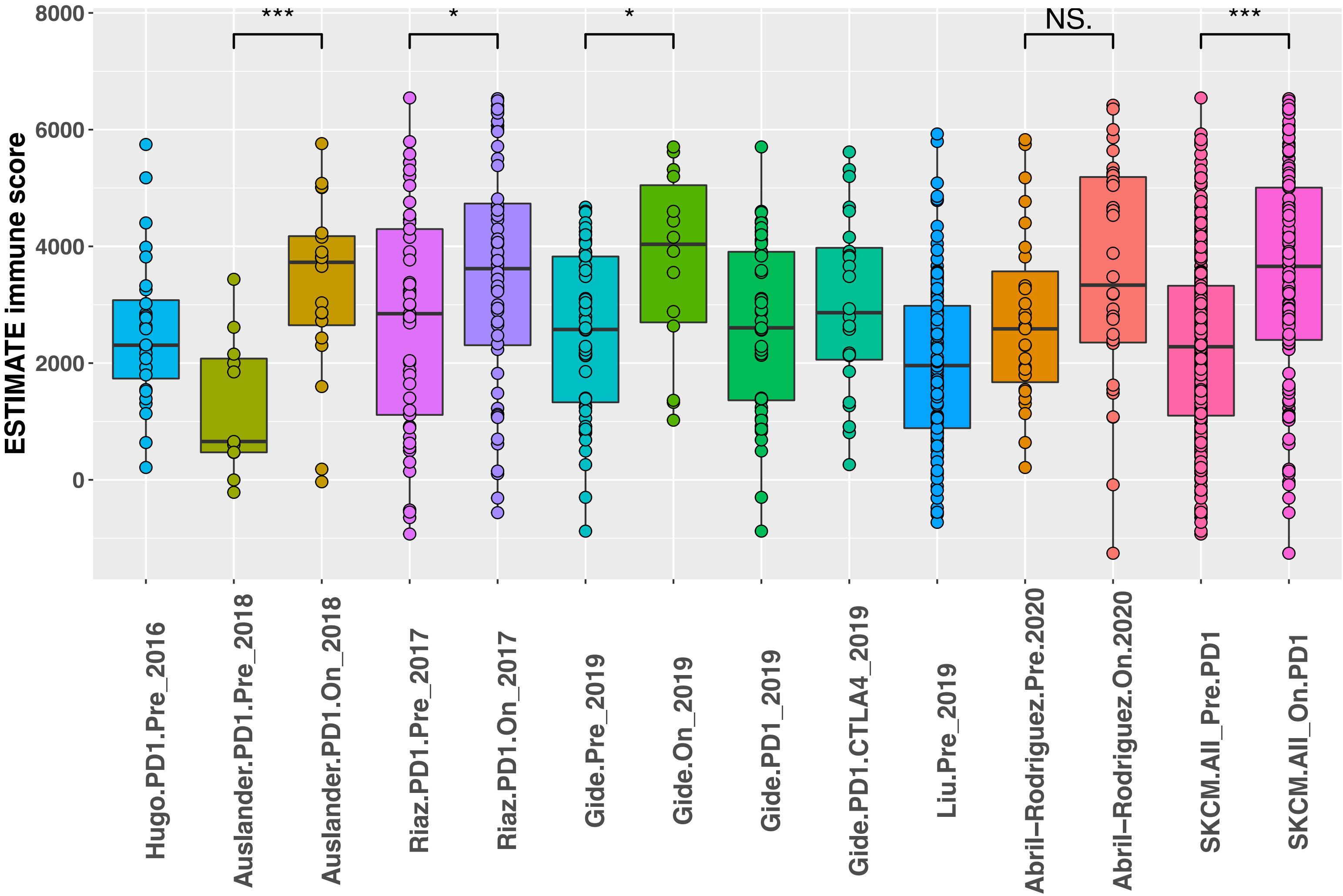

Supplementary Figure 7: **Boxplots of the ESTIMATE immune infiltration scores of samples across all the pre- and on-treatment datasets**. Boxplots show center line as the median, box limits as upper and lower quartiles of the data. The significances of the comparisons between pre- and on-treatment datasets from the same cohort were from the two-sides Wilcoxon rank sum test and are shown in asterisks (*** for p-value<0.001, ** for 0.001≤p-value<0.01, and * for 0.01≤p-value<0.05). The source data of this figure is provided in the Source Data file.

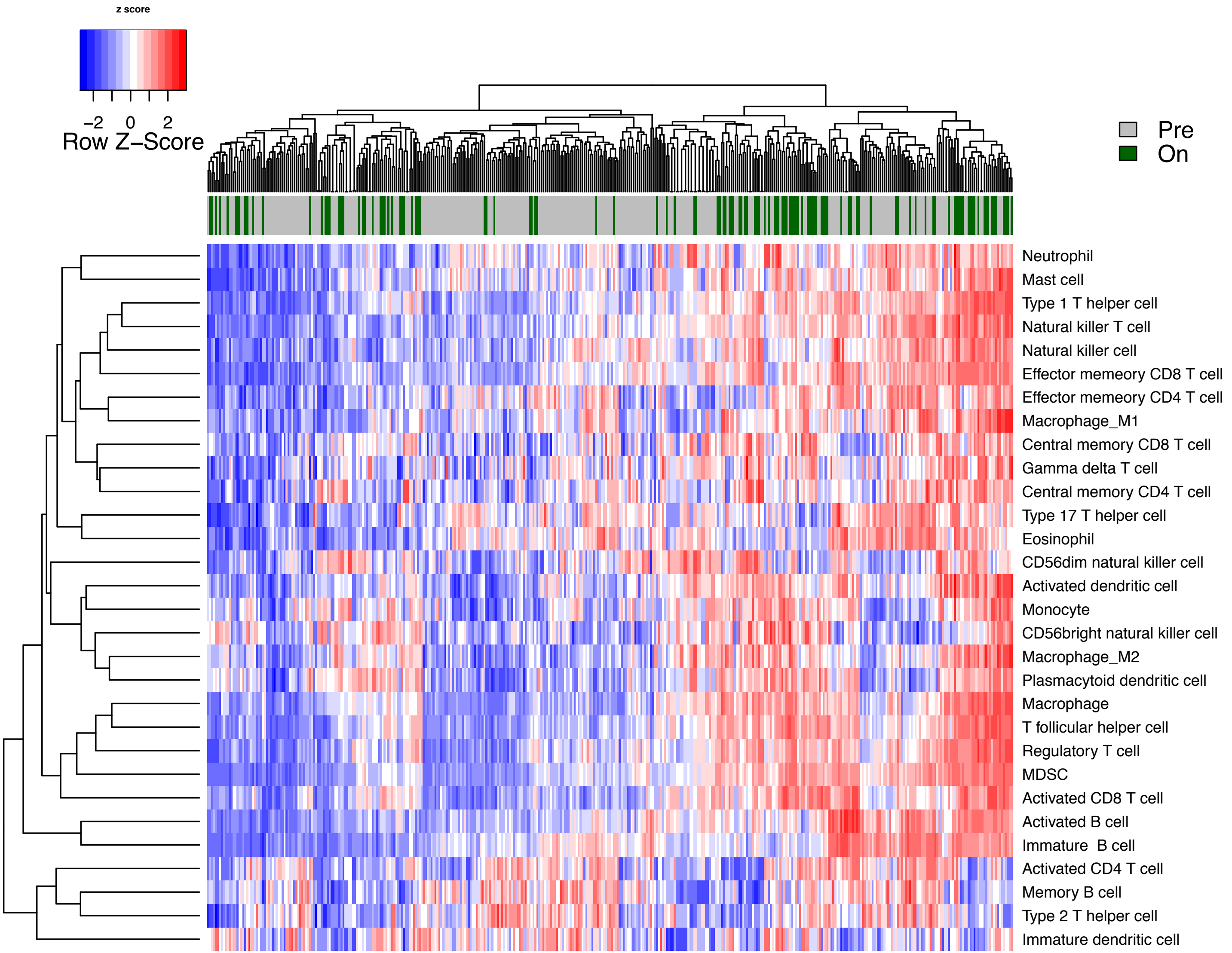

Supplementary Figure 8: **Immune cell profile of all the 411 pre- and on-treatment samples**. Immune cell profiles were characterized using single-sample GSEA scores of immune cell gene sets. The source data of this figure is provided in the Source Data file.

Supplementary Table 1: Performance of the MIAS-IMPRES predictors of SKCM patient response to anti-PD1 , evaluated using 5 fold cross-validation

	**Accuracy**	**Sensitivity**	**Specificity**
**Predictor for pre-treatment samples**	0.617±0.1	0.851±0.151	0.191±0.1
**Predictor for on-treatment samples**	0.81±0.045	0.894±0.049	0.614±0.139
