# Supplementary material for: Prediction of biomarkers and therapeutic combinations for anti-PD-1 immunotherapy using the global gene network association
Source: Nat Commun. 2022 Jan 10;13:42. doi: 10.1038/s41467-021-27651-4 (PMC8748689; doi:10.1038/s41467-021-27651-4)
Supplement: Supplementary file 3 — Description of Additional Supplementary Files [file 41467_2021_27651_MOESM3_ESM.pdf]

Description of Additional Supplementary Files

File Name: Supplementary Data 1
Description: MHC I network association prediction of all the genes in the gene network

File Name: Supplementary Data 2
Description: Selected top immune-positive and -negative genes in the 34 TCGA cancer types

File Name: Supplementary Data 3
Description:
a: Pathway enrichment analysis of the top immune-positive genes in the 34 TCGA cancer types
b: Pathway enrichment analysis of the top immune-negative genes in the 34 TCGA cancer types

File Name: Supplementary Data 4
Description: The top immune-positive genes shared by all TCGA cancer types

File Name: Supplementary Data 5
Description: Target genes of 36 compounds that were used for prediction evaluation in Figure 6.

File Name: Supplementary Data 6
Description: The top 100 immune-positve genes used for predicting SKCM patient response to anti-PD-1 therapies

File Name: Supplementary Data 7
Description: Patient information of the 354 pre- or on-treatment tumor samples used for evaluating predictions of anti-PD-1 therapy response
