# Supplementary material for: Prediction of biomarkers and therapeutic combinations for anti-PD-1 immunotherapy using the global gene network association
Source: Nat Commun. 2022 Jan 10;13:42. doi: 10.1038/s41467-021-27651-4 (PMC8748689; doi:10.1038/s41467-021-27651-4)
Supplement: Supplementary file 11 — Reporting Summary [file 41467_2021_27651_MOESM11_ESM.pdf]

nature portfolio

Corresponding author(s): Chia-Chin Wu; Y Alan Wang

Last updated by author(s): Nov 19, 2021

Reporting Summary

Nature Portfolio wishes to improve the reproducibility of the work that we publish. This form provides structure for consistency and transparency in reporting. For further information on Nature Portfolio policies, see our Editorial Policies and the Editorial Policy Checklist.

Statistics

For all statistical analyses, confirm that the following items are present in the figure legend, table legend, main text, or Methods section.

n/a	Confirmed	
☐	☒	The exact sample size (*n*) for each experimental group/condition, given as a discrete number and unit of measurement
☒	☐	A statement on whether measurements were taken from distinct samples or whether the same sample was measured repeatedly
☐	☒	The statistical test(s) used AND whether they are one- or two-sided<br>*Only common tests should be described solely by name; describe more complex techniques in the Methods section.*
☐	☒	A description of all covariates tested
☐	☒	A description of any assumptions or corrections, such as tests of normality and adjustment for multiple comparisons
☐	☒	A full description of the statistical parameters including central tendency (e.g. means) or other basic estimates (e.g. regression coefficient) AND variation (e.g. standard deviation) or associated estimates of uncertainty (e.g. confidence intervals)
☐	☒	For null hypothesis testing, the test statistic (e.g. *F*, *t*, *r*) with confidence intervals, effect sizes, degrees of freedom and *P* value noted<br>*Give P values as exact values whenever suitable.*
☒	☐	For Bayesian analysis, information on the choice of priors and Markov chain Monte Carlo settings
☒	☐	For hierarchical and complex designs, identification of the appropriate level for tests and full reporting of outcomes
☒	☐	Estimates of effect sizes (e.g. Cohen's *d*, Pearson's *r*), indicating how they were calculated

Our web collection on statistics for biologists contains articles on many of the points above.

Software and code

Policy information about availability of computer code

Data collection	Gene expression data, mutation calls and copy number segments of the 34 TCGA cancer types were also downloaded from the the Broad GDAC Firehose (https://gdac.broadinstitute.org/). The R package, CNTools was used to convert the segment data of the 34 TCGA cancer types into copy number of genes. The RNA-seq data of the 411 melnaoma samples were complied from the 6 published patient cohorts: Auslander (available in Gene Expression Omnibus: GSE115821), Gide (available in European Nucleotide Archive: PRJEB23709), Hugo (available in Gene Expression Omnibus: GSE78220), Liu (available in dbGaP: phs000452.v3.p1), Riaz (available in Gene Expression Omnibus: GSE91061), and Abril-Rodriguez (available in dbGaP: phs001919). RNA sequencing reads of the samples were mapped to the hg19 reference genome using the STAR aligner. For calculation of gene expression, raw count data of each gene were first obtained using with HTSeq(Anders et al. 2015) and were then converted to transcripts per million (TPM) that normalize counts for library size and gene length.
Data analysis	The data analysis was conducted by R version 4.0.2. The R script for calculating MIAS scores of melanoma samples is available in the GitHub repository (https://github.com/perwu/MIAS). The trained anti-PD1 response predictors for pre- and on-treatment melanoma patient samples are also available in the repository.

For manuscripts utilizing custom algorithms or software that are central to the research but not yet described in published literature, software must be made available to editors and reviewers. We strongly encourage code deposition in a community repository (e.g. GitHub). See the Nature Portfolio guidelines for submitting code & software for further information.

Data

Policy information about availability of data

All manuscripts must include a data availability statement. This statement should provide the following information, where applicable:

- Accession codes, unique identifiers, or web links for publicly available datasets
- A description of any restrictions on data availability
- For clinical datasets or third party data, please ensure that the statement adheres to our policy

Data Availability Statement

TCGA genomic and transcriptomic data used in this study are from the Broad Institute's Firehose data portal (https://gdac.broadinstitute.org/). The RNA-seq data of the 411 melnaoma samples were complied from the 6 published patient cohorts: Auslander (available in Gene Expression Omnibus: GSE115821), Gide (available in European Nucleotide Archive: PRJEB23709), Hugo (available in Gene Expression Omnibus: GSE78220), Liu (available in dbGaP: phs000452.v3.p1), Riaz (available in Gene Expression Omnibus: GSE91061), and Abril-Rodriguez (available in dbGaP: phs001919.v1.p1). The known target genes of compounds were compiled from the DGIdb database (https://www.dgidb.org/). The data underlying the figures are provided as a Source Data file. All the other data supporting the findings of this study are available within the article and its supplementary dataset and information files.

Field-specific reporting

Please select the one below that is the best fit for your research. If you are not sure, read the appropriate sections before making your selection.

☒ Life sciences ☐ Behavioural & social sciences ☐ Ecological, evolutionary & environmental sciences

For a reference copy of the document with all sections, see nature.com/documents/nr-reporting-summary-flat.pdf

Life sciences study design

All studies must disclose on these points even when the disclosure is negative.

Sample size	Not applicable, no experimental data. All data used in this study are from TCGA or prior published cohort studied.
Data exclusions	No data were excluded
Replication	Not applicable, no experimental data
Randomization	Not applicable, no experimental data
Blinding	Blinding not relevant in this study, no experimental data

Reporting for specific materials, systems and methods

We require information from authors about some types of materials, experimental systems and methods used in many studies. Here, indicate whether each material, system or method listed is relevant to your study. If you are not sure if a list item applies to your research, read the appropriate section before selecting a response.

Materials & experimental systems

n/a	Involved in the study	
☒	☐	Antibodies
☒	☐	Eukaryotic cell lines
☒	☐	Palaeontology and archaeology
☒	☐	Animals and other organisms
☒	☐	Human research participants
☒	☐	Clinical data
☒	☐	Dual use research of concern

Methods

n/a	Involved in the study	
☒	☐	ChIP-seq
☒	☐	Flow cytometry
☒	☐	MRI-based neuroimaging
